# Draft genomes of novel avian *Chlamydia abortus* strains from Australian Torresian crows (*Corvus orru*) shed light on possible reservoir hosts and evolutionary pathways

**DOI:** 10.1099/mgen.0.001134

**Published:** 2023-11-22

**Authors:** Vasilli Kasimov, Rhys T. White, Martina Jelocnik

**Affiliations:** ^1^​ University of the Sunshine Coast, School of Science, Engineering and Technology, Sippy Downs, Sunshine Coast, QLD 4556, Australia; ^2^​ Centre for Bioinnovation, University of the Sunshine Coast, Sippy Downs, Sunshine Coast, QLD 4557, Australia; ^3^​ Institute of Environmental Science and Research, Wellington, New Zealand

**Keywords:** *Chlamydia*, avian *Chlamydia abortus*, birds, Australia, culture-independent sequencing, multi-locus sequence typing (MLST), novel sequence type (ST)

## Abstract

*Chlamydia abortus,* an obligate intracellular bacterium, is a major causative agent of reproductive loss in ruminants, with zoonotic potential. Though this pathogen is primarily known to infect livestock, recent studies have detected and isolated genetically distinct avian strains of *C. abortus* from wild birds globally. Before this study, only five avian *C. abortus* genomes were publicly available. Therefore, we performed culture-independent probe-based whole-genome sequencing on clinical swabs positive for avian *C. abortus* obtained from Australian Torresian crows (*Corvus orru*) in 2019 and 2020. We successfully obtained draft genomes for three avian *C. abortus* strains (C1, C2 and C3), each comprising draft chromosomes with lengths of 1 115 667, 1 120 231 and 1 082 115 bp, and associated 7 553 bp plasmids, with a genome completeness exceeding 92 %. Molecular characterization revealed that these three strains comprise a novel sequence type (ST333), whilst phylogenetic analyses placed all three strains in a cluster with other avian *C. abortus* genomes. Interestingly, these three strains share a distant genomic relation (2693 single nucleotide variants) with the reference strain 15-58d/44 (ST152), isolated from a Eurasian magpie (*Pica pica*) in Poland, highlighting the need for more publicly available genomes. Broad comparative analyses with other avian *C. abortus* genomes revealed that the three draft genomes contain conserved *Chlamydia* genomic features, including genes coding for type III secretion system and polymorphic membrane proteins, and potential virulence factors such as the large chlamydial cytotoxin, warranting further studies. This research provides the first avian *C. abortus* draft genomes from Australian birds, highlighting Torresian crows as novel reservoir hosts for these potential pathogens, and demonstrates a practical methodology for sequencing novel *Chlamydia* genomes without relying on traditional cell culture.

## Data Summary

The study sequences are available in the National Center for Biotechnology Information (NCBI) under BioProject accession number PRJNA977824. Raw Illumina sequence read data generated in this study have been deposited in the NCBI sequence read archive (SRA; https://www.ncbi.nlm.nih.gov/sra) under accession numbers SRR24768373–SRR24768375. A complete list of SRA accession numbers is available in Table S7 (available in the online version of this article). The programs used to analyse raw sequence reads for whole-genome sequencing-based phylogenetic reconstruction are available as described in the Methods. The authors confirm that all supporting data, code and protocols have been provided in the article or supplementary data files.

**Impact Statement**

*
Chlamydia abortus* is an economically significant zoonotic pathogen that is exotic to Australia. This study advances the field by delivering the first draft genomes of avian *C. abortus* strains isolated from Australian Torresian crows, a newly identified reservoir host. Distinguished by a novel sequence type (ST333), these genomes expand the known genetic diversity of *C. abortus* and contain various potential virulence factors. Utilizing culture-independent probe-based whole-genome sequencing, this research provides a robust methodology for future chlamydial genomic studies without the reliance on laborious cell culture. Ultimately, this work broadens our understanding of avian *C. abortus* in a unique geographical and host context, whilst also raising pivotal questions regarding the pathogen’s global distribution, expanded host range and zoonotic potential.

## Introduction

Birds are recognized as long-distance vectors for various pathogens, including members of intracellular bacteria within the genus *Chlamydia *. These bacteria are globally distributed and infect a diverse range of animal hosts, with some species posing a zoonotic risk to humans. *Chlamydia psittaci* has long been the predominant species causing avian chlamydiosis and zoonosis. However, over the past two decades, global studies have shown that birds can also harbour other genetically diverse chlamydial species, including an emerging lineage of avian *Chlamydia abortus* strains. These strains have since been detected globally in wild bird families, including Anatidae, Corvidae, Psittacidae, Phylloscopidae, Rallidae, Sylviidae and Diomeidae [1–5]. Initially, researchers classified these previously unclassified *Chlamydia* spp. as atypical *C. psittaci* or *C. psittaci */* C. abortus* intermediates, based on their genomic similarities to both *C. psittaci* and *C. abortus* species [6]. However, subsequent genomic analyses of these strains, including the previously classified *C. psittaci* strain 84/2334 from a yellow-crowned amazon parrot (*Amazona ochrocephala*), demonstrated a closer relationship to traditional livestock *C. abortus* strains and they have since been reclassified as avian *C. abortus* strains [1, 6]. In addition, the avian strains of *C. abortus* are distinguished from livestock strains by the presence of a conserved chlamydial 7.5 kb plasmid and chlamydial cytotoxin gene (*tox*B) in their plasticity zones, features not present in livestock *C. abortus* genomes. Based on these findings, the authors recommend that the current taxonomic definition of *C. abortus* includes both avian and classical livestock isolates [1, 6].

In our previous study, we provided the initial molecular characterizations of avian *C. abortus* strains from wild Australian birds [7], raising serious concerns, as the traditional livestock *C. abortus* is considered exotic in Australia and New Zealand [8]. Although these strains differ from those detected in livestock, their pathogenicity and spillover potential to humans, livestock and other animals remains uncertain. Therefore, this study aimed to further characterize these avian strains using culture-independent whole-genome sequencing (WGS) to evaluate their phylogenetic relationships with other global avian and livestock isolates and genome content. In doing so, we provide the first draft genomes of three avian *C. abortus* strains from Australian Torresian crows (*Corvus orru*) as novel reservoir hosts and offer new insights into the genetic diversity of the avian *C. abortus* clade.

## Methods

### Sample descriptions used for whole-genome sequencing

During our previous study [7], eye, choanal and cloacal swabs were taken from three Torresian crows (strains C1, C2 and C3) admitted to the Australian Zoo Wildlife Hospital (Beerwah, Queensland, Australia) due to unspecified trauma and clinical disease during 2019 and 2020. These swabs were processed and screened using a *Chlamydiaceae *-specific quantitative (q)PCR assay, followed by preliminary molecular characterization of the full-length 16S rRNA and *omp*A genes, to confirm chlamydial infection and the genetic identity of these infecting organisms (as described in our previous study) [7].

### Avian *C. abortus* probe-based capture using SureSelect targeted enrichment and Illumina MiSeq DNA sequencing

WGS using probe-capture was performed utilizing a set of 120-mer biotinylated RNA probes designed with the assistance of Agilent Technologies using the Tier2 design (0.5–2.99 Mb). The probes were designed to cover the complete chromosome (including the consensus sequence of conserved and unique polymorphic regions) and plasmid of six reference genomes (Table S1). These include *C. abortus* strains: 84/2334 (GenBank: CP031646); 15-58d44 (GenBank: GCA_905143135); 15-49d3 (GenBank: GCA_900416705); 15-70d24 (GenBank: GCA_900416725) (Table S2), *Chlamydia buteonis* strain IDL17-4553 (GenBank: CP050318) (Table S3) and *C. psittaci* strain Horse_Pl (GenBank: CP025423) (Table S4). The libraries targeting avian *Chlamydia* from the three DNA samples were generated in the Australian Genome Research Facility (Parkville, Victoria, Australia) using Agilent SureSelect XT HS2 library preparation with an Agilent SureSelect XT HS Enzymatic fragmentation kit as per the manufacturer’s instructions. Subsequent bait capture was completed with the Agilent SureSelect Capture Custom Probe kit as per the manufacturer’s instructions, where the biotinylated RNA probes and SureSelectXT reagents were added to the DNA library, hybridizing with the targeted chlamydial DNA. Before WGS, magnetic separation was performed utilising streptavidin-coated beads to separate the hybridzed DNA from the remaining complex DNA mixture, as previously described [9].

Following capture of the DNA libraries, their quality was assessed using the Agilent TapeStation D1000 assay and qPCR. Subsequently, the libraries were normalized and pooled, and three libraries were sequenced as 2×150 bp paired-end reads on the MiSeq platform using V2-300 chemistry (Illumina) at the Australian Genome Research Facility. All sequence data from three samples passed quality control, as they exhibited a high proportion of chlamydial reads, had ≥20× sequence coverage and were selected for further phylogenetic analyses.

### Quality control, read mapping and draft genome assembly

The tools within the Qiagen CLC Genomics Workbench v.22.0.0 platform (https://digitalinsights.qiagen.com/) were used with default parameters to assess the sequence read data quality, remove Illumina adaptor sequences and failed reads, and filter on read length (≤100 and ≥200 bp) for each sample. Using default settings, the trimmed and filtered paired reads were mapped to the complete chromosome and plasmid of the *C. abortus* reference strain 84/2334 (GenBank: CP031646). The filtered paired reads were also assembled into *de novo* contigs utilizing SPAdes v3.13.0 [10] as implemented in the online Bacterial and Viral Bioinformatics Resource Center (BV-BRC) genome assembly tool [11], using a minimum contig coverage and length threshold of 10× and 300 bp, respectively. *De novo* contigs were annotated using RAST-tk v.1.3.0 [12], as implemented in BV-BRC. Lastly, WebQUAST (http://cab.cc.spbu.ru/quast/) [13], an online server for quality assessment and comparison of genome assemblies based on the QUAST tool [14], was used to evaluate our genome assembly and completeness, using *C. abortus* strain 84/2334 as the reference genome (Table 1). Average nucleotide identity (ANI) was calculated using CJ Bioscience’s online ANI calculator [15] utilizing the OrthoANIu algorithm with default parameters for comparison of *de novo* contigs of strains C1, C2 and C3 and selected avian chlamydial genomes (Table S5). For each strain, sequence data were compiled into a single FASTQ file for each of the forward and reverse reads. These were then submitted to the National Center for Biotechnology Information (NCBI) sequence read archive (SRA) under the BioProject accession number PRJNA977824.

**Table 1.** Descriptions and draft genome metrics of avian *C. abortus* strains C1, C2 and C3

Strain	Feature	Value
**C1**		
Strain descriptions	MLST/*omp*A genotype	ST333/6N
	Collection year	2019
	Location	Queensland, Australia
	Host	Torresian crow
	Clinical presentations	Unwell
	Anatomical site	Eye/choana
Chromosome	No. of total reads (paired)	2 828 134
	Mean read length (bp)	150
	No. of total trimmed reads	2 702 297 (95.55 %)
	No. of mapped trimmed reads to 84/2334	2 638 807 (97.65 %)
	Av. coverage (sd)	317.25 (±168.45)
	No. of *de novo* contigs	14
	Total length (bp)	1 115 667
	Mean depth (short reads)	242.31
	Mean short-read coverage	299.85
	Contig N50 value (bp)	183 552
	Draft chromosome G+C content (%)	39.84
	Genome completeness (%)	95.07
Plasmid	No. of *de novo* contigs	1
	Total length (bp)	7553
	Mean coverage	163.27
	Plasmid G+C content (%)	33.00
**C2**		
Strain descriptions	MLST/*omp*A genotype	ST333/A
	Collection year	2019
	Location	Queensland, Australia
	Host	Torresian crow
	Clinical presentations	Unwell
	Anatomical site	Eye/choana/cloaca
Chromosome	No. of total reads (paired)	3 032 333
	Mean read length (bp)	150
	No. of total trimmed reads	2 622 501 (86.48 %)
	No. of mapped trimmed reads to 84/2334	2 202 761 (85.72 %)
	Av. coverage (sd)	252.22 (±143.85)
	No. of *de novo* contigs	13
	Total length (bp)	1 120 231
	Mean depth (short reads)	118.21
	Mean short-read coverage	205.50
	Contig N50 value (bp)	197 835
	Draft chromosome G+C content (%)	39.84
	Genome completeness (%)	95.86
Plasmid	No. of *de novo* contigs	1
	Total length (bp)	7553
	Mean coverage	352.58
	Plasmid G+C content (%)	33.00
**C3**		
Strain descriptions	MLST/*omp*A genotype	ST333/6N
	Collection year	2020
	Location	Queensland, Australia
	Host	Torresian crow
	Clinical presentations	Trauma
	Anatomical site	Eye/choana/cloaca
Chromosome	No. of total reads (paired)	2 446 888
	Mean read length (bp)	150
	No. of total trimmed reads	1 851 374 (75.66 %)
	No. of mapped trimmed reads to 84/2334	1 233 879 (68.94 %)
	Av. coverage (sd)	146.29 (±330.46)
	No. of *de novo* contigs	16
	Total length (bp)	1 082 115
	Mean depth (short reads)	46.80
	Mean short-read coverage	128.80
	Contig N50 value (bp)	197 835
	Draft chromosome G+C content (%)	39.86
	Genome completeness (%)	92.63
Plasmid	No. of *de novo* contigs	1
	Total length (bp)	7553
	Mean coverage	54.81
	Plasmid G+C content (%)	33.00

### Taxonomic classification of the three avian-derived genomes

**Fig. 1.**
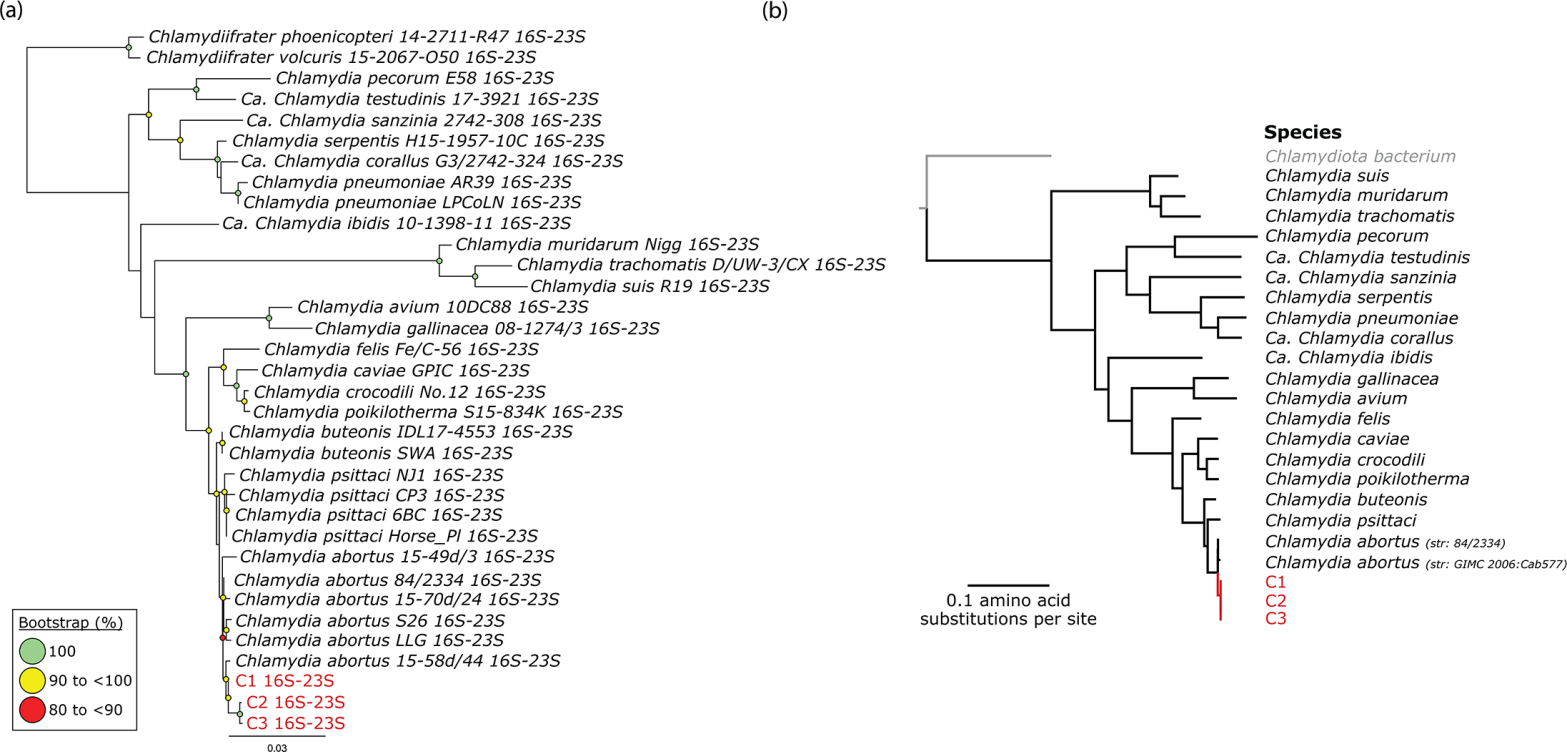
Taxonomic identification of novel avian *Chlamydia abortus* strains. (**a**) The mid-point rooted maximum-likelihood phylogenetic analysis of the complete 16S–23S rRNA gene sequences of the three avian *C. abortus* strains from this study and 31 representative *Chlamydiaceae* strains. Bar, 0.03 nucleotide substitutions per site. Bootstrap values >80 % (using 1000 replicates) are shown. (**b**) The maximum-likelihood approximation was reconstructed using a concatenated alignment of 105 conserved bacterial markers. Taxonomy is shown according to the GTDB (s_, species) from the NCBI taxonomy. The tree is rooted according to the outgroup *Chlamydiota bacterium*; bar, 0.1 amino acid substitutions per site. Red type represents the strains sequenced in this study for both trees.

Before initial phylogenetic analyses and strain characterization, the complete 16S–23S rRNA operon genes were extracted from the draft genomes sequenced in this study from all three samples (C1, C2 and C3). The complete 16S rRNA gene, 23S rRNA gene and 16S–23S rRNA operon sequences from the three samples in this study and 31 publicly available strains representative of the family *Chlamydiaceae* were aligned using MAFFT v7.450 [16] (as implemented in Geneious Prime v2023.0.4; https://www.geneious.com), producing a 1 559, 2 961 and 4 778 bp alignment, respectively. The best-fit model, determined using the Bayesian Information Criterion (BIC) for all three alignments using ModelFinder [17], is the Time-Variable Model with empirical base frequencies, consideration of invariant sites and a discrete gamma distribution with four categories (TVM+F+I+G4). A mid-point rooted maximum-likelihood phylogenetic tree was reconstructed using IQ-TREE2 v2.1.2(1 000 bootstrap replicates) [18] (Fig. 1a). Individual 16S rRNA and 23S rRNA phylogenetic trees were also reconstructed (using the methods outlined above) (Fig. S1).

The draft assemblies of genomes C1, C2 and C3 were also analysed using the Genome Taxonomy Database Toolkit (GTDB-Tk) genome-based taxonomy (GTDB-Tk v2.1.1 with GTDB package R207_v2 [19, 20]) to determine their closest taxonomic neighbours. A concatenated reference alignment of 105 bacterial marker genes was constructed using only sequences from the genus *Chlamydia* (g_, genus) extracted from the GTDB-Tk. The taxonomic tree was inferred using maximum-likelihood approximation with FastTree v2.1.7 [21] under the WAG model [22] of protein evolution with gamma-distributed rate heterogeneity [23] (+GAMMA) (Fig. 1b). The accession numbers for each strain included in these analyses (Fig. 1) are available in Table S6.

### Variant detection and phylogenetic analyses

**Fig. 2.**
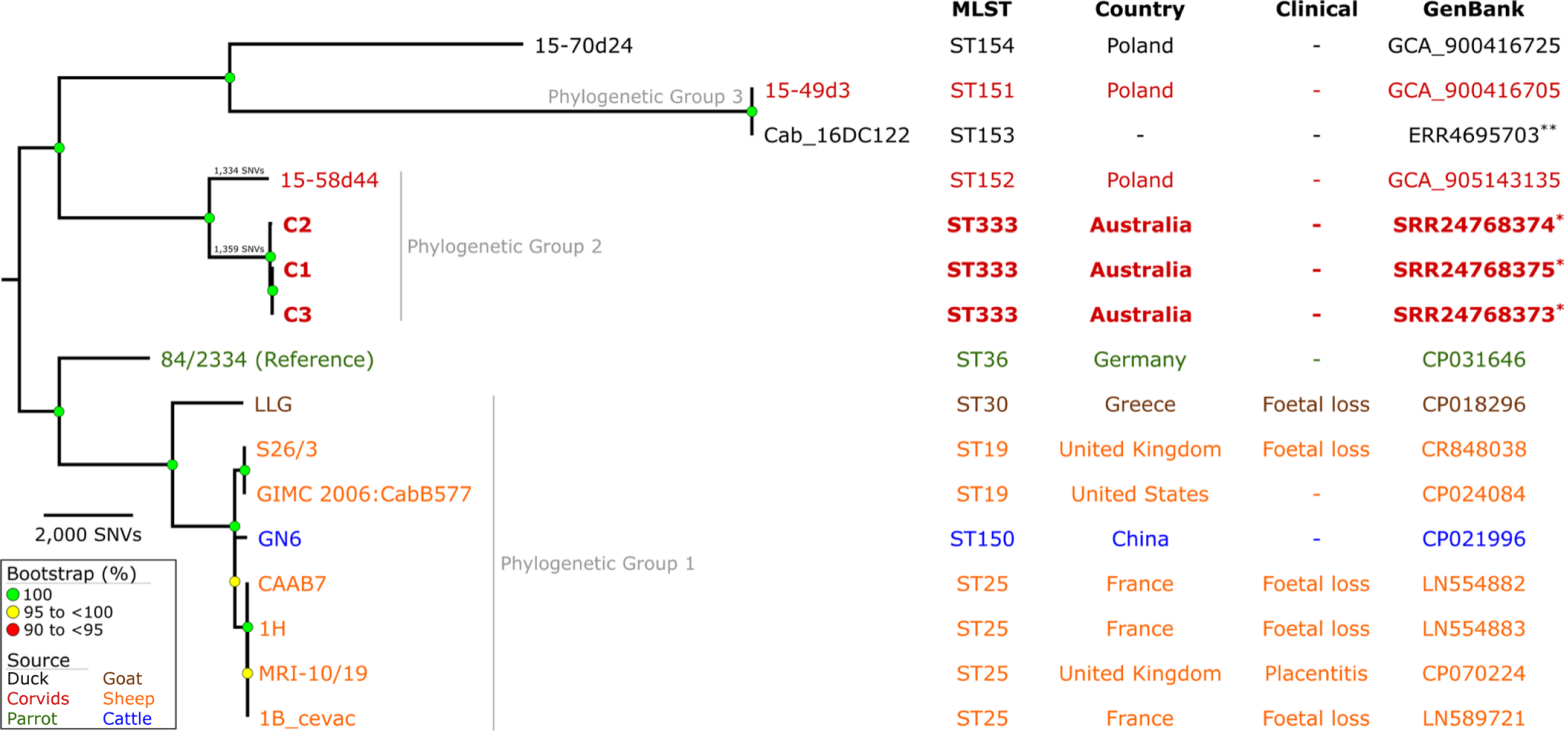
Variant detection and phylogenetic analyses of *C. abortus* strains. Maximum-parsimony tree reconstructed using a core-genome SNV alignment of three strains from this study and 13 *C. abortus* reference genomes, using 1000 bootstrap replicates. Strains are coloured according to their isolated hosts, with strains from this study being in bold type. Bootstrap values >90 % are shown, with branch length displaying the number of SNVs compared to the reference strain (84/2334). Single asterisks (*) indicate sequence read data generated in this study and uploaded to the NCBI sequence read archive. Double asterisks (**) indicate sequence read data downloaded from the NCBI sequence read archive and were assembled in this study.

The genomes of C1, C2 and C3 were compared to 13 global and publicly available *C. abortus* genomes from other sources to estimate the number of single-nucleotide variants (SNVs) and determine their phylogenetic relationships (Table S7). Parsnp v1.7.4 [24] was used to generate a core-genome alignment (alignment of the syntenic regions across all genomes), using the genome of *C. abortus* strain 84/2334 as a reference. When using 84/2334 as a reference for calling SNVs, 35 642 SNVs were identified across the 1 056 122 bp core-genome alignment. The resulting SNV alignment was used to reconstruct phylogenetic maximum-parsimony trees using the heuristic search feature of PAUP v4.0a [25] before adding 1000 bootstrap replicates. The resulting phylogenetic trees were visualized using FigTree v1.4.4 (http://tree.bio.ed.ac.uk/software/figtree/, accessed on 10 March 2023) (Fig. 2). To further identify robust Phylogenetic Groups (PGs) within each phylogeny, each alignment file was put into rhierBAPS v1.0.1 (an R [26] implementation of hierarchical Bayesian Analysis of Population Structure, BAPS) [27] with one level of clustering, allowing up to 20 initial clusters.

### Phylogenetic analysis of plasmid sequences

All three complete chlamydial plasmid sequences from the strains in this study were aligned against other publicly available reference plasmid sequences (avian *C. abortus *, *C. psittaci* and *C. buteonis*) using MAFFT v7.450 (as implemented in Geneious Prime v2023.0.4). The best-fit model, determined using the BIC using ModelFinder, is the Kimura three-parameter model with unequal purine transitions, empirical base frequencies and invariant sites (K3Pu+F+I). A mid-point rooted maximum-likelihood phylogenetic tree was reconstructed using IQ-TREE2 v2.1.2(1 000 bootstrap replicates) (Fig. S2).

### Multi-locus sequence typing and *omp*A genotyping

Metadata for all strains included in multi-locus sequence typing (MLST) and *omp*A phylogenetic analyses are described in Table S8. The *omp*A and *Chlamydiales* MLST (*gat*A, *gid*A, *eno*A, *hem*N, *hlf*X and *opp*A) genes were extracted from the draft genomes sequenced in this study from all three samples (C1, C2 and C3).

**Fig. 3.**
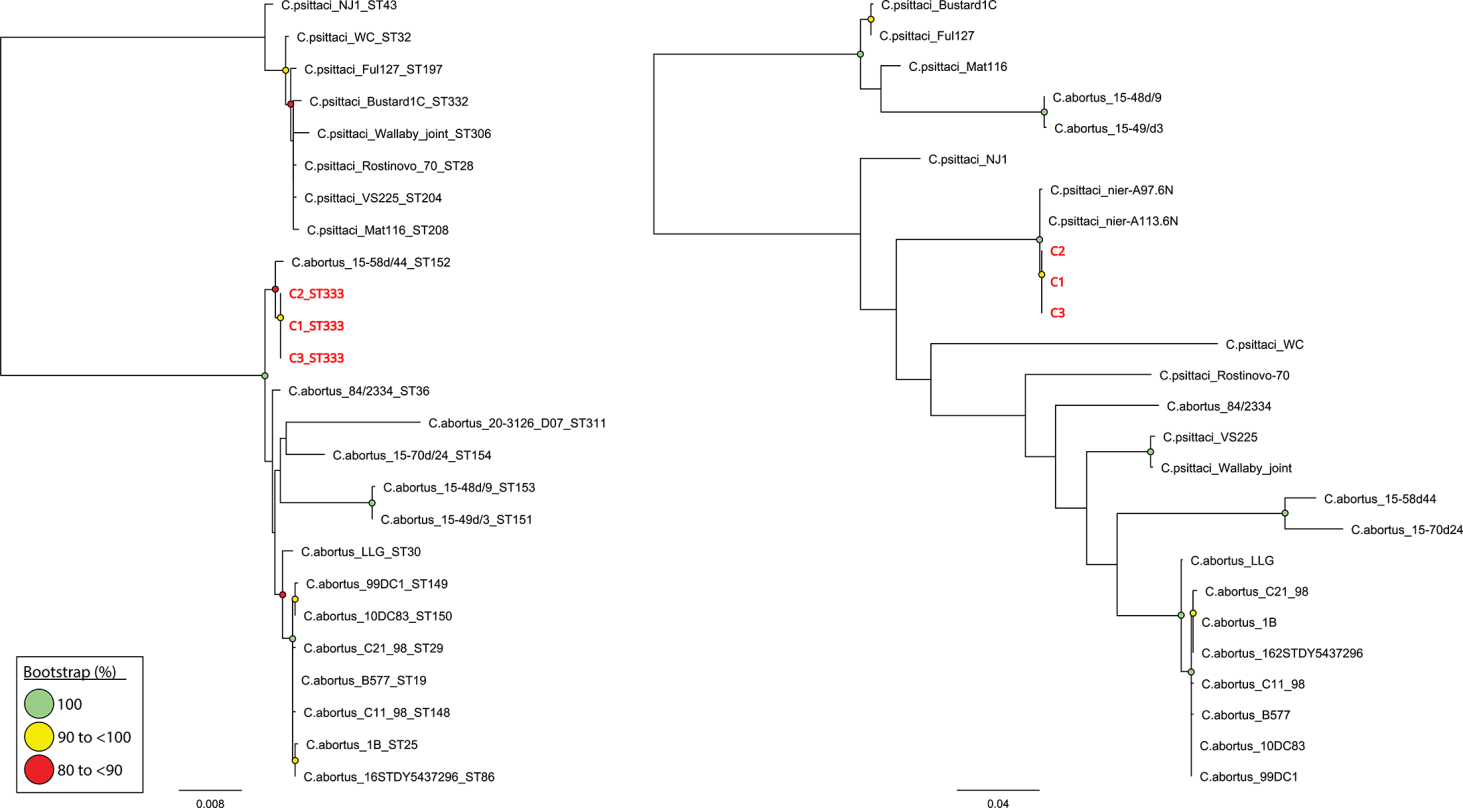
Maximum-likelihood phylogenetic analysis of (**a**) 3 098 bp alignment representing concatenated MLST sequences, including three from this study and 22 reference sequences, and (**b**) 1 215 bp alignment of *omp*A sequences, including three from this study and 23 reference sequences. Both phylogenies are midpoint rooted. Branch lengths represent nucleotide substitutions per site. Bootstrap values (1000 replicates) >80 % are shown. All three strains from this study are coloured in red.

The MLST genes from all three samples in this study were concatenated and aligned against 22 publicly available *C. abortus* and *C. psittaci* reference sequence types (STs) using MAFFT (as implemented in Geneious Prime v2023.0.4), producing a 3 098 bp alignment. The best-fit model, determined using the BIC using ModelFinder, is the Kimura three-parameter model with unequal purine transitions, empirical base frequencies and invariant sites (K3Pu+F+I). A mid-point rooted maximum-likelihood phylogenetic tree was reconstructed using IQ-TREE2 v2.1.2 (1 000 bootstrap replicates) (Fig. 3a). The MLST gene sequences and STs from the draft genomes sequenced in this study were deposited in the publicly available *Chlamydiales* database on PubMLST under strain IDs: 5009, 5010 and 5011 [28].

Similarly, the three *omp*A sequences from this study and 23 publicly available *C. abortus* and *C. psittaci* reference *omp*A sequences were also aligned using MAFFT (as implemented in Geneious Prime v2023.0.4), producing a 1 215 bp alignment. The best-fit model, determined using the BIC using ModelFinder, is the Hasegawa–Kishino–Yano model with empirical base frequencies and a discrete gamma distribution with four categories (HKY+F+G4). A mid-point rooted maximum-likelihood phylogenetic tree was reconstructed using IQ-TREE2 v2.1.2(1 000 bootstrap replicates) (Fig. 3b).

Lastly, MLST and *omp*A sequences from 24 strains (three from this study and 21 reference strains with paired MLST and *omp*A sequences) were concatenated and aligned using MAFFT (as implemented in Geneious Prime v2023.0.4), producing a 4 398 bp nucleotide alignment. The best-fit model, determined using the BIC using ModelFinder, is the two-parameter model with unequal rates, empirical base frequencies, invariant sites and a discrete gamma distribution with four categories (TPM3u+F+I+G4). A mid-point rooted maximum-likelihood phylogenetic tree was reconstructed using IQ-TREE2 v2.1.2(1 000 bootstrap replicates) (Fig. S3).

## Results

### Culture-independent WGS and draft-genome quality assessment

We aimed to use newly designed custom capture probes to sequence the genomes of novel avian *C. abortus* strains (preliminarily identified based on 16S rRNA and *omp*A genotyping) detected in Australian Torresian crows. WGS using new custom-designed baits targeting avian *Chlamydia* genomes resulted in all three samples being successfully sequenced and draft genomes (covering both chromosome and plasmid) produced. The sequence read data and *de novo* contigs quality metrics are summarized in Table 1.

The mapping of short reads from strains C1, C2 and C3 to the complete chromosome and plasmid of *C. abortus* reference strain 84/2334 indicates an average chromosome coverage >238-fold and plasmid coverage >190-fold, with relatively even coverage across both for all three samples (Table 1). *De novo* assembly of trimmed paired reads from the three avian *C. abortus* strains from this study produced draft chromosome lengths of 1 115 667, 1 120 231 and 1 082 115 bp for strains C1, C2 and C3, respectively, with a median GC content of 39.85 %. All three draft *de novo* assemblies demonstrate a high level of completeness compared to *C. abortus* reference strain 84/2334, with the highest level of completeness shown in strain C2 (95.86 %), followed by C1 (95.07 %) and C3 (92.57 %). Lastly, *de novo* assembly produced a complete 7 553 bp contiguous sequence of the chlamydial plasmid from all three samples.

### Phylogenetic analysis of the 16S–23S rRNA operon and taxonomic identification

The taxonomic position of the three avian *C. abortus* strains from this study within the family *Chlamydiaceae* was determined by phylogenetic tree reconstruction of the individual 16S and 23S rRNA genes, complete 16S–23S rRNA operon and 105 conserved bacterial markers, demonstrating that they all belong to the genus *Chlamydia* and species *C. abortus* (Figs 1 and S1). More specifically, all three strains belong to the avian *C. abortus* lineage, as they all cluster in the same clade with strain 15-58d/44, isolated from a Eurasian magpie (*Pica pica*) in Poland (Figs 1 and S1). The 16S–23S rRNA operon of strain C1 displayed the highest sequence pairwise identity (99.83 %) to this reference strain, followed by C2 (99.47 %) and C3 (99.45 %) 16S–23S rRNA operon sequences, respectively (Fig. 1a).

Furthermore, the ANI was also calculated for all three draft genomes in comparison to other closely related avian *C. abortus* reference genomes (15-58d/44, 15-70d/24, 15-49d/3 and 84/2334). For all three strains, avian *C. abortus* reference strain 15-58/44 produced the highest nucleotide similarity values (ANI) of 99.58, 99.58 and 99.59 % when compared against strains C1, C2 and C3, respectively, further confirming species identity (ANI >95 %) (Table S5).

### Genomic diversity of *C. abortus* strains from Australian Toressian crows

To further assess the genomic diversity of the three avian *C. abortus* strains from this study, a maximum-parsimony phylogenetic tree was reconstructed from an alignment of 35 642 core-genome SNVs from the three genomes from this study and 13 other publicly available *C. abortus* reference genomes (Fig. 2). Most clades had a bootstrap value of 100%, highlighting the robustness of the evolutionary inferences made. All three strains from our study clustered within a distinct sub-clade with avian *C. abortus* strain 15-58d/44, forming PG2. Other avian *C. abortus* strains formed distinct lineages (82/2334 and 15-70d/24), whilst strains 15-49d/3 and Cab_16DC122 formed a separate clade (PG3). All traditional livestock *C. abortus* strains resolved into a genetically distinct sister clade, forming PG1 (Fig. 2).

The assembled plasmid sequences from all three strains (pC1, pC2 and pC3) clustered in a well-supported subclade with pCab_15-58d/44 from the closely related avian *C. abortus* 15-58d/44 and, within a broader clade, also containing pCab_84/2334. Sequences pC2 and pC3 were identical to each other, sharing a 99.87 % nucleotide identity to pCab_15-58d/44, whilst pC1 was 99.96 % identical to pC2 and pC3, sharing 99.83 % similarity to p15-58d/44 (Fig. S2). The plasmids from the other two avian *C. abortus* strains (pCab 15-49d/3 and pCab 15-70d/24) clustered into their own genetically distinct clade.

### Assessing genetic diversity of avian *C. abortus* strains with MLST and *omp*A genotyping

Due to its widespread use in genetic diversity studies and where WGS is not available, we also applied MLST and *omp*A genotyping to classify strains (C1, C2 and C3) from this study within the broader *C. abortus* population. Using MLST, all three samples resolved as a novel ST333, clustering in a distinct sub-clade with ST152 (avian *C. abortus* strain 15-58d/44) (Fig. 3a), congruent with the core-genome SNV tree shown in Fig. 2. Other avian *C. abortus* STs (ST36, ST311, ST154, ST153 and ST151) resolved into their own second distinct genomically distinct sub-clade, while livestock *C. abortus* STs formed a third distinct yet closely related sub-clade (Fig. 3a). All *C. psittaci* STs formed their own genetically distinct clade.

Next, we wanted to evaluate genetic diversity using *omp*A genotyping. The *omp*A sequences from the three samples in this study were 100 % identical to each other, whilst clustering into a distinct clade with *omp*A genotype 6N from *C. psittaci* strains nier_A113 and nier_A97, respectively (Fig. 3b). The C1, C2 and C3 *omp*A sequences shared 99.90 and 99.79% nucleotide similarity with the *omp*A genotype 6N. The *omp*A sequences from closely related avian *C. abortus* strains [84/2334 (*omp*A genotype F), 15-49/d3 and 15-48d/9 (*omp*A genotype G2)] clustered into other genetically diverse clades and lineages with *C. psittaci omp*A sequences. The *omp*A sequences from avian 15-70d/24 (*omp*A genotype G1) and 15-58d/44 (genotype 1V) strains formed their own distinct sub-clade, within a larger clade, including livestock *C. abortus omp*A sequences (Fig. 3b).

Due to contrasting phylogenetic clustering of strains using different gene markers, and in an attempt to provide further phylogenetic resolution, we performed phylogenetic analysis of concatenated MLST and *omp*A sequences for 24 *C. abortus* and *C. psittaci* strains, where we had paired *omp*A and MLST sequences. In these analyses, all eight *C. psittaci* and 16 avian and livestock *C. abortus* strains formed their own distinct phylogenetic groups. The *C. abortus* strains resolved into two distinct genetically diverse sub-clades. The three strains from this study formed into a distinct monophyletic lineage (ST333/genotype 6N) within the first sub-clade that also includes avian *C. abortus* 84/2334, 15-49d/3 and 15-58d/9 strains (Fig. S3). Other closely related avian *C. abortus* (15-58d/44 and 15-70d/24) strains formed a distinct lineage within a second larger genetically diverse sub-clade, also including all livestock *C. abortus* strains (Fig. S3).

### Gene variation between avian *C. abortus* strains

This study also broadly compared all three new draft genomes against four reference avian *C. abortus* strains (15-58d/44, 15-70d/24, 15-49d/3 and 84/2334) to determine whether any hallmark chlamydial chromosomal elements, including genes coding for polymorphic membrane proteins (*pmp*s), inclusion membrane proteins (*incs*), the type III secretion system (T3SS), tryptophan operon (*trp*) and proteins in the plasticity zone are present (Table S9) [29]. The *de novo* contigs of C1, C2 and C3 strains reveal 11, 10 and 12 intact or partial *pmp* genes, respectively. Additionally, 32 intact T3SS genes were detected in all three strains, encoding structural proteins, chaperones and effectors, along with up to four intact or partial *inc* genes. As expected, the *trp* operon (*trp*ABFCDR) and complementary genes (*kyn*U, *prs*A) were absent in all three strains, echoing the gene profiles of other avian *C. abortus* reference strains. Despite the incomplete assembly of the plasticity zone, the *de novo* contigs of all three strains contained the outer-boundary biotin modification genes (*accB* and *accC*), which are present in all avian *C. abortus* reference genomes, except strain 15-58d/44. Furthermore, sequences coding for a single (partial) copy of the large cytotoxin (*toxB*) gene, which is intact in all reference genomes, were identified from all strains from this study. Strains C2 and C3 also appear to harbour genes for IMP dehydrogenase (similar to strains 15-70d/24 and 15-58d/44) and GMP synthase (unique to strain 15-58d/44), though these genes were inconclusive in the draft genome of C1 (Table S9). Other genes identified within the plasticity zone encoded hypothetical proteins with undefined functions, and no phospholipase D (*PLD*) genes were observed. Given that the strains identified in this study are incomplete draft assemblies, it is important to note that the precise composition and number of *incs*, *pmps*, T3SS genes, and genes within the plasticity zone remain to be resolved.

## Discussion

This study provides the first draft genomes of genetically diverse avian *C. abortus* strains from Australia, further confirming that common avian species in Australia, such as Torresian crows, may act as reservoir hosts for these potential pathogens. Molecular characterization demonstrated that these strains are a novel ST333. The most recent common ancestor to ST333 is genomically related to the avian *C. abortus* genome 15-58d/44 (ST152 from a Eurasian magpie in Poland). However, a substantial pairwise SNV distance of 2693 SNVs indicates that ST333 and ST152 belong to distinct populations and suggests a more distant historical divergence from a common ancestor further in the past. To gain a more comprehensive insight and a clearer perspective into genomic diversity, it would be valuable to sequence more ST152 and ST333 genomes from more individuals.

Following our previous study, where we initially detected this avian subspecies [7], we utilized an increasingly popular method involving culture-independent probe-based WGS of DNA extracted from dry swabs [9]. Relying on only the preliminary molecular characterization of these strains, we applied a novel custom probe design based on several reference genomes from closely related chlamydial species in an attempt to resolve draft genomes. The three draft genomes from this study resolved with high completeness (≥92 %), providing the first valuable insights into the genetic diversity of Australian *C. abortus* strains. Although costly, custom-designed probe-based capture has resulted in more successful WGS attempts than traditional methods [9]. For example, a previous study also using *C. psittaci* probe-based capture obtained full genome coverage, despite some read sets only containing ~12 % of chlamydial reads [30].

A key limitation of this study was the inability to sequence and accurately resolve highly polymorphic regions, including *incs*, *pmps* and the plasticity zone genes [1]. To address this issue, it is recommended that future studies utilize additional sequencing methods, such as Oxford Nanopore Technologies (ONT) nanopore sequencing, coupled with a short-read probe-capture WGS approach (particularly when uncertain about the correct reference genome to utilize). Whilst the same designed RNA probes can be used to capture the target DNA, it is imperative to proceed with a specialized library preparation tailored for ONT sequencing post-capture. This involves ligating ONT-specific adapters to the DNA fragments, a crucial step facilitating passage through nanopores for sequencing. Unlike short-read technologies, ONT is capable of generating reads spanning several kilobases, thus facilitating the sequencing of highly polymorphic regions that may be otherwise difficult to resolve. Over time, the provision of an expanded catalogue of chlamydial genomes from traditional and newly characterized species will enable a more tailored, successful and cost-efficient custom probe design for future chlamydial studies.

Broad comparison to other avian *C. abortus* genomes revealed that all three strains from this study also carry a conserved 7.5 kb chlamydial plasmid and indicate the presence of the large chlamydial cytotoxin (*tox*B) gene in the plasticity zone – features not present in traditional livestock *C. abortus* strains [31]. Although the exact function of these genes within these avian *C. abortus* strains is still to be elucidated, there is increasing evidence that the chlamydial plasmid plays a vital role in the virulence, persistent infection and pathogenicity in other infecting strains [29]. Therefore, the presence of a chlamydial plasmid within these avian *C. abortus* strains prompts future plasmid-based transformation studies to investigate gene function and the potential role these plasmids contribute to host/tissue tropism. Moreover, the inclusion of the virulence-associated *tox*B gene within these and other avian *C. abortus* strains may also provide insights into the potential pathogenicity of these species, particularly as the predicted protein products cluster with those identified in virulent *C. psittaci* (ST24/genotype A) strains [6]. Although studies investigating chlamydial *tox*B functionality are lacking, *tox*B orthologues identified in *Escherichia coli* and *Citrobacter rodentium* are capable of Ras superfamily inactivation and host cytoskeleton disassembly; additionally, they are hypothesized to play a similar role in chlamydial species [32]. Similar to other chlamydial genomes, an increased number of polymorphisms were identified in several *pmps*, *inc*s and four T3SS effector genes. In *C. psittaci* genomes, these polymorphic regions are hypothesized to contribute to differences in virulence and host tropisms [32–34]. More avian *C. abortus* genomes (supplemented with cell biology studies) will be needed to elucidate the role of these genes in virulence and host tropisms.

Phylogenetic analyses of the 16S–23S rRNA operon, core-genome, MLST and *omp*A genes reveal that these strains comprise a novel ST333, have a known *omp*A genotype (6N) and are most similar to avian *C. abortus* strains detected in European avian species. Interestingly, reference strain 15-58d/44, detected from a Eurasian magpie (family Corvidae), shares the highest degree of genetic similarity to all three novel strains in this study, showcasing the association between genotypes 1V and 6N and corvid species, such as crows and Eurasian magpies [1, 6, 35–37]. It is worth noting that while MLST was congruent with the core-genome SNV phylogeny, the genetic diversity using the *omp*A gene yielded conflicting phylogenetic clustering for avian *C. abortus *. In several instances, the *omp*A sequences from avian *C. abortus* strains were more similar to those from *C. psittaci *, which may lead to taxonomic misclassification of the infecting strains [35]. Therefore, concatenating MLST and *omp*A sequences (in the absence of WGS) may provide fine-detailed molecular characterization of infecting chlamydial strains. The analysis of concatenated MLST and *omp*A genes further demonstrated the genetic distinctiveness of these strains, as denoted by their novel ST and unique *omp*A genotype. However, this is probably due to the unavailability of MLST sequences from samples identified as *omp*A genotypes 1V and 6N, identified in other global Corvidae species [38, 39]. Thus, performing MLST (and, where possible, WGS) on these strains is crucial for elucidating their phylogenetic positioning, potentially aligning them with the three strains described in the current study. Global studies have unveiled other genetically diverse avian *C. abortus* STs, such as ST311, identified from waved albatross (*Phoebastria irrorata*) in South America, and ST151, ST153 and ST154, detected from Polish wildfowl. It is plausible that these global lineages also include other avian *C. abortus */* C. psittaci* intermediates, which have presently not been whole-genome sequenced or multi-locus sequence typed [1, 35, 38–40].

Based on the findings of this current research and our previous study, such avian *C. abortus* lineages are probably actively circulating within wild Australian bird populations, infecting a broad range of avian (and potentially other) hosts. Although the clinical nature of these strains remains undetermined, it may be practical to assume that these strains may be pathogenic and have zoonotic potential due to being genetic intermediates of two pathogens of veterinary and economic significance (livestock *C. abortus* and *C. psittaci* strains).

## Conclusion

This study provides a practical and novel methodology for the WGS of novel *Chlamydia* without the reliance on cell culture. However, in cases where the appropriate reference is not readily identified, this methodology may benefit from integration with ONT nanopore sequencing to bridge significant gaps within highly polymorphic regions of the chlamydial genome. Furthermore, this study provides the first Australian avian *C. abortus* draft genomes collected from Torresian crows, highlighting these species as novel reservoir hosts. The molecular data indicate that these avian *C. abortus* strains comprise a novel sequence type (ST333) and exhibit a degree of similarity to avian *C. abortus* strain 15-58d/44, found in a Polish Eurasian magpie. Lastly, the substantial pairwise SNV distances between the three strains from this study and reference genomes highlight a significant gap in the existing literature and an urgent need for more genomes and surveillance studies to identify more closely related strains and uncover the full host range of this potential pathogen.

## Supplementary Data

Supplementary material 1

Supplementary material 2

## References

[1] Zaręba-Marchewka K, Szymańska-Czerwińska M, Livingstone M, Longbottom D, Niemczuk K (2021). Whole genome sequencing and comparative genome analyses of *Chlamydia abortus* strains of avian origin suggests that *Chlamydia abortus* species should be expanded to include avian and mammalian subgroups. Pathogens.

[2] Zaręba-Marchewka K, Szymańska-Czerwińska M, Niemczuk K (2020). Chlamydiae - What’s new?. J Vet Res.

[3] Stokes HS, Berg ML, Bennett ATD (2021). A review of chlamydial infections in wild birds. Pathogens.

[4] Aaziz R, Vinueza RL, Vorimore F, Schnee C, Jiménez-Uzcátegui G (2023). Avian *Chlamydia abortus* strains detected in galápagos waved albatross (*Phoebastria irrorata*). J Wildl Dis.

[5] Van Loock M, Vanrompay D, Herrmann B, Vander Stappen J, Volckaert G (2003). Missing links in the divergence of *Chlamydophila abortus* from *Chlamydophila psittaci*. Int J Syst Evol Microbiol.

[6] Longbottom D, Livingstone M, Ribeca P, Beeckman DSA, van der Ende A (2021). Whole genome de novo sequencing and comparative genomic analyses suggests that *Chlamydia psittaci* strain 84/2334 should be reclassified as *Chlamydia abortus* species. BMC Genomics.

[7] Kasimov V, Dong Y, Shao R, Brunton A, Anstey SI (2022). Emerging and well-characterized chlamydial infections detected in a wide range of wild Australian birds. Transbound Emerg Dis.

[8] Jelocnik M (2019). *Chlamydiae* from down under: the curious cases of chlamydial infections in Australia. Microorganisms.

[9] White RT, Anstey SI, Kasimov V, Jenkins C, Devlin J (2022). One clone to rule them all: culture-independent genomics of *Chlamydia psittaci* from equine and avian hosts in Australia. Microb Genom.

[10] Prjibelski A, Antipov D, Meleshko D, Lapidus A, Korobeynikov A (2020). Using SPAdes de novo assembler. Curr Protoc Bioinformatics.

[11] Olson RD, Assaf R, Brettin T, Conrad N, Cucinell C (2023). Introducing the Bacterial and Viral Bioinformatics Resource Center (BV-BRC): a resource combining PATRIC, IRD and ViPR. Nucleic Acids Res.

[12] Brettin T, Davis JJ, Disz T, Edwards RA, Gerdes S (2015). RASTtk: a modular and extensible implementation of the RAST algorithm for building custom annotation pipelines and annotating batches of genomes. Sci Rep.

[13] Mikheenko A, Saveliev V, Hirsch P, Gurevich A (2023). WebQUAST: online evaluation of genome assemblies. Nucleic Acids Res.

[14] Gurevich A, Saveliev V, Vyahhi N, Tesler G (2013). QUAST: quality assessment tool for genome assemblies. Bioinformatics.

[15] Yoon SH, Ha SM, Lim J, Kwon S, Chun J (2017). A large-scale evaluation of algorithms to calculate average nucleotide identity. Antonie van Leeuwenhoek.

[16] Nakamura T, Yamada KD, Tomii K, Katoh K (2018). Parallelization of MAFFT for large-scale multiple sequence alignments. Bioinformatics.

[17] Kalyaanamoorthy S, Minh BQ, Wong TKF, von Haeseler A, Jermiin LS (2017). ModelFinder: fast model selection for accurate phylogenetic estimates. Nat Methods.

[18] Minh BQ, Schmidt HA, Chernomor O, Schrempf D, Woodhams MD (2020). IQ-TREE 2: new models and efficient methods for phylogenetic inference in the genomic era. Mol Biol Evol.

[19] Parks DH, Chuvochina M, Waite DW, Rinke C, Skarshewski A (2018). A standardized bacterial taxonomy based on genome phylogeny substantially revises the tree of life. Nat Biotechnol.

[20] Chaumeil PA, Mussig AJ, Hugenholtz P, Parks DH (2022). GTDB-Tk v2: memory friendly classification with the genome taxonomy database. Bioinformatics.

[21] Price MN, Dehal PS, Arkin AP (2009). FastTree: computing large minimum evolution trees with profiles instead of a distance matrix. Mol Biol Evol.

[22] Whelan S, Goldman N (2001). A general empirical model of protein evolution derived from multiple protein families using a maximum-likelihood approach. Mol Biol Evol.

[23] Yang Z (1994). Maximum likelihood phylogenetic estimation from DNA sequences with variable rates over sites: approximate methods. J Mol Evol.

[24] Treangen TJ, Ondov BD, Koren S, Phillippy AM (2014). The Harvest suite for rapid core-genome alignment and visualization of thousands of intraspecific microbial genomes. Genome Biol.

[25] Wilgenbusch JC, Swofford D (2003). Inferring evolutionary trees with PAUP*. Curr Protoc Bioinformatics.

[26] RStudio Team (2020). RStudio: Integrated Development Environment for R.

[27] Tonkin-Hill G, Lees JA, Bentley SD, Frost SDW, Corander J (2018). RhierBAPS: an R implementation of the population clustering algorithm hierBAPS. Wellcome Open Res.

[28] Jolley KA, Bray JE, Maiden MCJ (2018). Open-access bacterial population genomics: BIGSdb software, the PubMLST.org website and their applications. Wellcome Open Res.

[29] Luu LDW, Kasimov V, Phillips S, Myers GSA, Jelocnik M (2023). Genome organization and genomics in *Chlamydia*: whole genome sequencing increases understanding of chlamydial virulence, evolution, and phylogeny. Front Cell Infect Microbiol.

[30] Branley J, Bachmann NL, Jelocnik M, Myers GSA, Polkinghorne A (2016). Australian human and parrot *Chlamydia psittaci* strains cluster within the highly virulent 6BC clade of this important zoonotic pathogen. Sci Rep.

[31] Seth-Smith HMB, Busó LS, Livingstone M, Sait M, Harris SR (2017). European *Chlamydia abortus* livestock isolate genomes reveal unusual stability and limited diversity, reflected in geographical signatures. BMC Genomics.

[32] Sachse K, Hölzer M, Vorimore F, Barf L-M, Sachse C (2023). Genomic analysis of 61 *Chlamydia psittaci* strains reveals extensive divergence associated with host preference. BMC Genomics.

[33] Dean D, Weil MR, Frace M, MacCannell D, Srinivasamoorthy G (2015). *Chlamydia psittaci* comparative genomics reveals intraspecies variations in the putative outer membrane and type III secretion system genes. Microbiology.

[34] Favaroni A, Trinks A, Weber M, Hegemann JH, Schnee C (2021). Pmp repertoires influence the different infectious potential of avian and mammalian *Chlamydia psittaci* strains. Front Microbiol.

[35] Szymańska-Czerwińska M, Mitura A, Niemczuk K, Zaręba K, Jodełko A (2017). Dissemination and genetic diversity of chlamydial agents in Polish wildfowl: Isolation and molecular characterisation of avian *Chlamydia abortus* strains. PLoS One.

[36] Zaręba-Marchewka K, Szymańska-Czerwińska M, Mitura A, Niemczuk K (2019). Draft genome sequence of avian *Chlamydia abortus* genotype G1 strain 15-70d24, isolated from Eurasian Teal in Poland. Microbiol Resour Announc.

[37] Zaręba-Marchewka K, Szymańska-Czerwińska M, Niemczuk K (2021). Draft genome sequences of avian *Chlamydia abortus* genotype G2 strain 15-49d3, isolated from Mallard, and genotype 1V strain 15-58d44, isolated from Magpie in Poland. Microbiol Resour Announc.

[38] Stalder S, Marti H, Borel N, Sachse K, Albini S (2020). Occurrence of *Chlamydiaceae* in raptors and crows in Switzerland. Pathogens.

[39] Jeong J, An I, Oem J-K, Wang S-J, Kim Y (2017). Molecular prevalence and genotyping of *Chlamydia* spp. in wild birds from South Korea. J Vet Med Sci.

[40] Stalder S, Marti H, Borel N, Vogler BR, Pesch T (2021). Falcons from the United Arab Emirates infected with *Chlamydia psittaci*/*C abortus* intermediates specified as *Chlamydia buteonis* by polymerase chain reaction. J Avian Med Surg.

