# Supplementary material for: Draft genomes of novel avian Chlamydia abortus strains from Australian Torresian crows (Corvus orru) shed light on possible reservoir hosts and evolutionary pathways
Source: Microb Genom. 2023 Nov 22;9(11):001134. doi: 10.1099/mgen.0.001134 (PMC10711307; doi:10.1099/mgen.0.001134)
Supplement: Supplementary material 1 [file mgen-9-1134-s001.pdf]

SUPPLEMENTARY APPENDIX

# Draft genomes of novel avian *Chlamydia abortus* strains from Australian Torresian Crows (*Corvus orru*) shed light on possible reservoir hosts and evolutionary pathways


## Author names

Vasilli Kasimov[1,2*], Rhys T. White[3] and Martina Jelocnik[1,2]

## Affiliation

[1] University of the Sunshine Coast; School of Science, Engineering and Technology, Sippy Downs, Sunshine Coast, QLD 4557, Australia

[2] Centre for Bioinnovation, University of the Sunshine Coast, Sippy Downs, Sunshine Coast, QLD 4556, Australia.

[3] Institute of Environmental Science and Research, Wellington, New Zealand.

## Corresponding author

[*] Corresponding author: Vasilli Kasimov, University of the Sunshine Coast, Centre for Bioinnovation, Sippy Downs, Queensland 4556, Australia.

Email: vasilli.kasimov@research.usc.edu.au



## Keywords

*Chlamydia*; avian *Chlamydia abortus*; crows; Australia; culture-independent sequencing; multi-locus sequence typing (MLST); novel sequence type (ST)

**<u>This file includes the following:</u>**

**Supplementary Materials, Figure S1:** Traditional taxonomic identification of novel chlamydial strains.

**Supplementary Materials, Figure S2:** Chlamydial plasmid comparisons between closely related species.

**Supplementary Materials, Figure S3:** Phylogenetic analysis of concatenated MLST and *omp*A sequences.

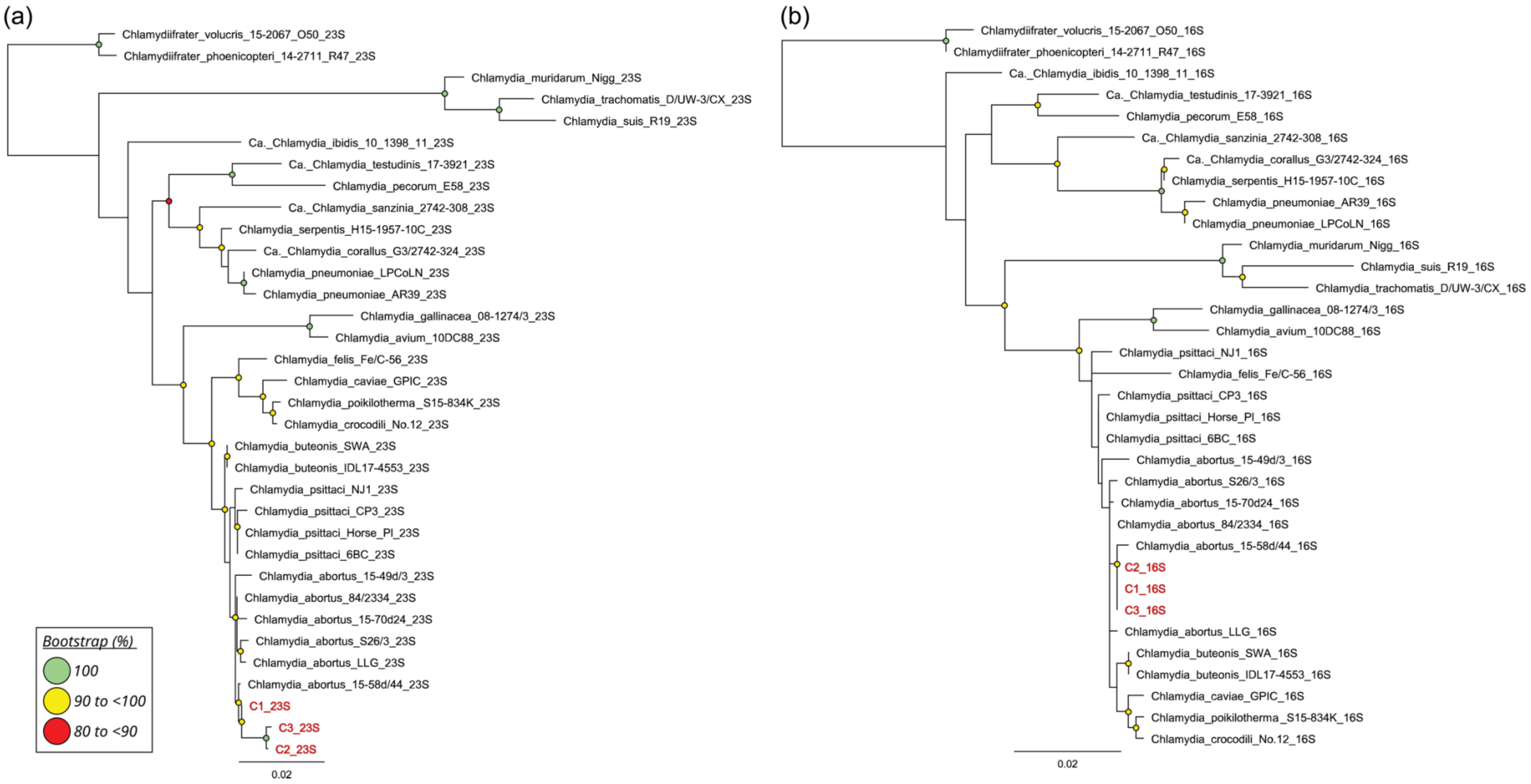


**Figure S1: Traditional taxonomic identification of novel chlamydial strains.** ML phylogenetic trees constructed from the (a) 2,961 bp alignment of the chlamydial 23S rRNA gene, and (b) 1,559 bp alignment of the chlamydial 16S rRNA gene from the three strains in this study and 31 publicly available strains representative of the family *Chlamydiaceae* using IQTREE2. Branch lengths represent the nucleotide substitutions per site. Bootstrap values greater than 80% (using 1,000 replicates) are shown.

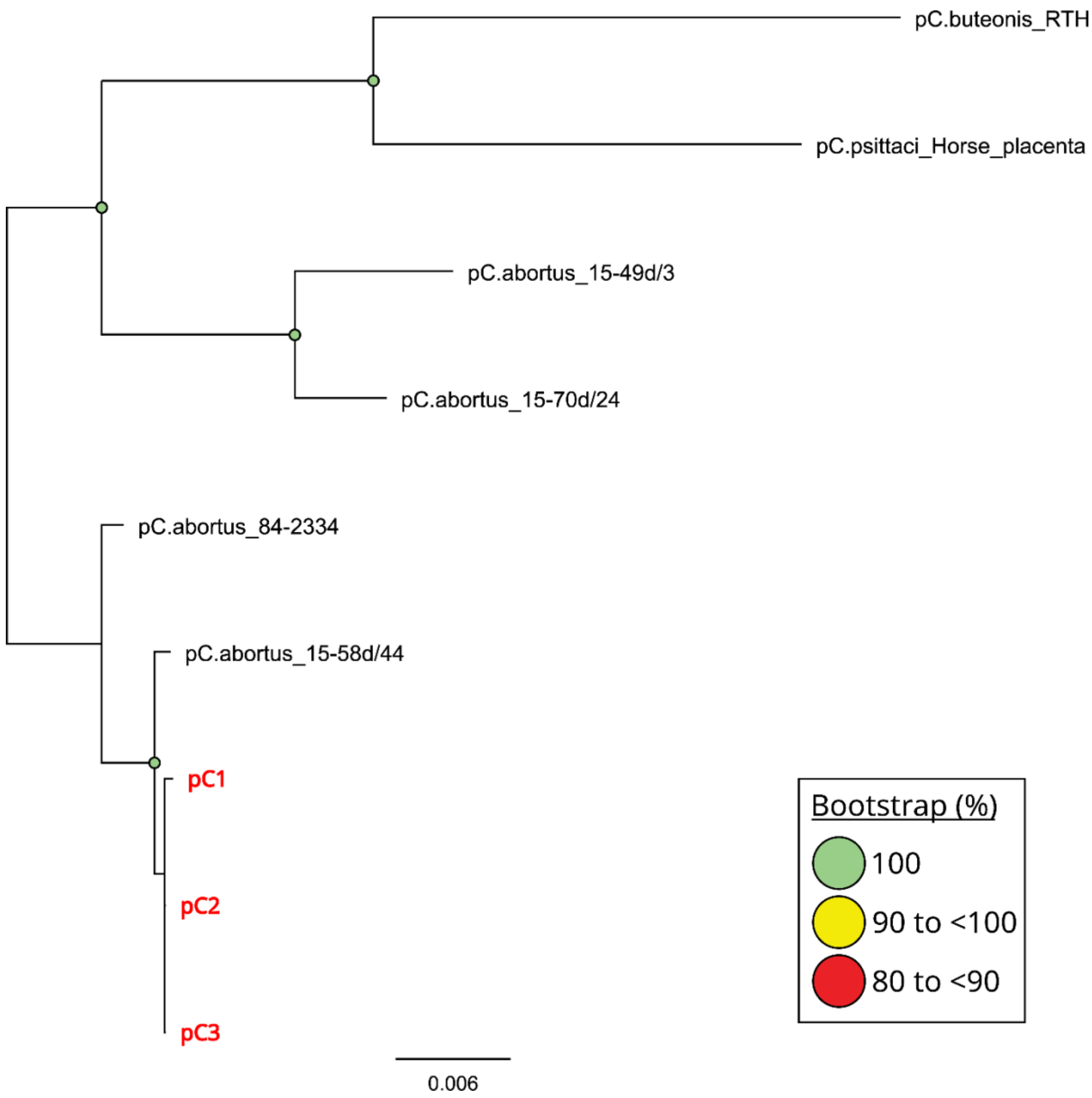


**Figure S2: Chlamydial plasmid comparisons between closely related species.** A ML phylogenetic tree was constructed from the three *de novo assembled* plasmids in this study and six chlamydial reference plasmids. The phylogenetic tree was constructed from a 7,562 bp MAFFT alignment using IQTREE2. Bootstrap values greater than 80% (using 1,000 replicates) are shown.

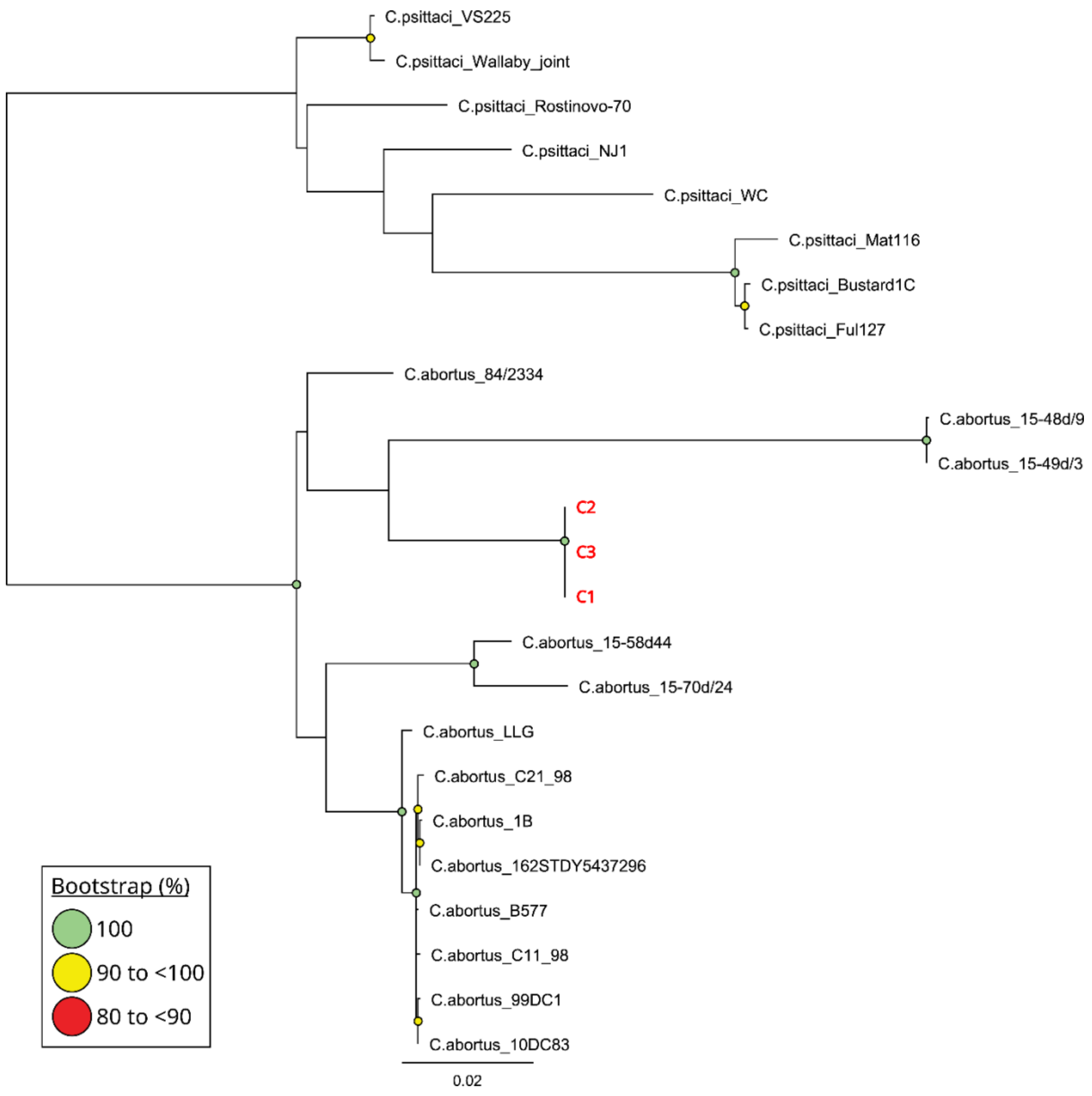


**Figure S3: Phylogenetic analysis of concatenated MLST and *ompA* sequences.** A ML phylogenetic tree constructed from a 4,564 bp alignment of 24 concatenated MLST and *omp*A sequences (three from this study and 21 reference sequences) using IQTREE2. As indicated by the scale bar, branch lengths represent the nucleotide substitutions per site. Bootstrap values (1,000 replicates) greater than 80% are shown.
